# Change in postgraduate medical education – how much didactic shaping is possible at all? A document analysis of the guideline regulations on specialty training 1992-2018 with a focus on surgery

**DOI:** 10.3205/zma001723

**Published:** 2024-11-15

**Authors:** Sarah Prediger, Daniela Rastetter, Sigrid Harendza

**Affiliations:** 1 University Medical Center Hamburg-Eppendorf, III Medical Clinic, Hamburg, Germany; 2 University of Hamburg, Faculty of Economics and Social Sciences, Hamburg, Germany

**Keywords:** specialty training, postgraduate education, surgery, didactics

## Abstract

**Background:**

The structures of postgraduate medical education are regulated by the (guideline) regulations on specialty training ((M-)WBO). This formal structure is the result of medical discourse between medical associations, specialist societies and other associations. Various developments can be seen in the WBO. This study examines whether changes at the level of the WBO can contribute to changing and didactically optimizing postgraduate education in hospitals.

**Methods:**

Based on Mayring's theory a document analysis of the MWBO 1992, 2003 and 2018 was carried out with an additional focus on aspects of surgery. For this purpose, texts and contents of the MWBOs were compared and word frequencies were analyzed. In addition, three guided interviews with experts were conducted and analyzed according to Kuckartz using MAXQDA. The experts were selected based on their position and their involvement in the MWBO adaptation process.

**Results:**

The analysis of the WBO shows that efforts are being made to adapt specialty training in hospitals in order to make it more structured and didactically optimized. Concepts are being introduced and, in some cases abolished (e.g. specialist knowledge “Fachkunden”) or further developed (e.g. competencies). The word frequency analysis shows the use of the same eight most frequent terms, which seem to define the basic character of postgraduate education. There are also obstacles to communication between the medical self-administration and the clinical stakeholders, which limit the possibilities for change.

**Conclusion:**

For implementation of the WBO in hospitals, clinicians need to be included even more intensively. This should not only take place during the development of the WBO, but especially during its implementation in the hospitals themselves to enable better integration of the new structures and didactic concepts of the WBO.

## Zusammenfassung

**Hintergrund:**

Die Strukturen der fachärztlichen Weiterbildung werden durch die (Muster-)Weiterbildungsordnung ((M-)WBO) geregelt. Diese formale Strukturierung entsteht im ärztlichen Diskurs zwischen Ärztekammern, Fachgesellschaften und weiteren Verbänden. Dabei zeigen sich verschiedene Entwicklungen in den WBO. In dieser Arbeit wird untersucht, inwiefern Veränderungen auf Ebene der WBO dazu beitragen können, die Weiterbildung in den Kliniken zu verändern und didaktisch zu optimieren.

**Methodik:**

Es wurde eine Dokumentenanalyse der MWBO 1992, 2003 und 2018 mit ergänzendem Fokus auf Aspekte der Chirurgie theoriegeleitet nach Mayring durchgeführt. Hierfür wurden Texte und Inhalte der MWBO verglichen sowie die Worthäufigkeiten analysiert. Ergänzend wurden drei leitfadengestützte Expert:innen-Interviews geführt und inhaltsanalytisch nach Kuckartz mit MAXQDA ausgewertet. Die Auswahl der Expert:innen erfolgte qua Position und ihrer Beteiligung am Anpassungsprozess der MWBO.

**Ergebnisse:**

In der Analyse der WBO zeigen sich Anpassungsbestrebungen, die Weiterbildung in den Kliniken strukturierter und didaktisch optimierter zu gestalten. Es werden Konzepte eingeführt und teilweise wieder abgeschafft (z.B. Fachkunden) oder weiterentwickelt (z.B. Kompetenzen). Die Worthäufigkeitsanalyse zeigt die Nutzung der gleichen acht häufigsten Begriffe, die den grundlegenden Charakter der Weiterbildung zu definieren scheinen. Es zeigen sich außerdem Kommunikationshindernisse zwischen der ärztlichen Selbstverwaltung und den klinischen Akteur:innen, die Veränderungsmöglichkeiten einschränken.

**Schlussfolgerung:**

Für die Umsetzung der WBO in den Kliniken bedarf es einer noch intensiveren Ansprache der Kliniker:innen. Dies sollte nicht nur bei der Ausarbeitung der WBO erfolgen, sondern besonders bei deren Implementierung in den Kliniken selbst, um die neuen Strukturen und didaktischen Konzepte der WBO besser intergieren zu können.

## 1. Background

Postgraduate medical education is an essential part of the professional socialization process of physicians. In Germany, it is designed as “learning on the job” and takes place during clinical work. Physicians in postgraduate education are also employees in a hospital and are defined as employees who receive their specialty training while working, i.e. on the side. The structures of postgraduate education are regulated by the specialty training regulations (“Weiterbildungsordnung”, WBO) and additional training guidelines (“Weiterbildungsrichtilinien”, WB-RiLi). This formal structuring is the result of medical discourse at the level of the State Chambers of Physicians, specialist societies (“Fachgesellschaften”, FG) and other medical associations and is thus organized and implemented by the medical profession itself. Initially, guideline regulations on specialty training (“Musterweiterbildungsordnung”, short: MWBO) are drawn up in this process, which, following its adoption by the German Medical Assembly (“Deutscher Ärztetag”, DÄT), is transposed into state law with the publication of a state-specific WBO by the relevant State Chamber of Physicians (“Landesärztekammer”, LÄK). In the following, the term MWBO is used when explicitly referring to the MWBO document, otherwise the generally used term WBO is used.

A total of nine WBOs were published between 1924 and 2018. Looking at the historical development of the structuring of specialty training in the shape of the WBO, three processes can be identified:

1. differentiation,
2. structuring and
3. didacticization [1].

The first regulation, known as the “Facharztordnung”, was adopted at the 43^rd^ DÄT in 1924 with the title: “Guidelines for the recognition and practical activities of medical specialists”. The reason for the first guidelines was a long discussion about specialization in medicine and the *“full doctor”* (Vollarzt) status (in the sense of one physician is able to handle all diseases) can established at the time [2], [3], [4]. However, the latter was gradually replaced by a progressive specialization process. In the following years, the development of the WBO was characterized by a process of differentiation in which more specialist titles were steadily defined. In 1968, now under the title “Weiterbildungsordnung”, the concept of subspecialization was introduced and a division into areas, sub-areas and additional designations was implemented for the first time. These changes were initiated, on the one hand, because signs of division were emerging between the major disciplines of surgery and internal medicine [4], and, on the other hand, because the content and boundaries of the subspecialties needed to be more clearly defined [2]. As a supplement to the WBO, WB-RiLi were also issued for the first time at this time as *“guidelines”* for trainers, trainees and the State Chambers of Physicians. The WB-RiLi set out in detail what knowledge and skills had to be taught in specialty [4].

The introduction of examinations for specialist diploma with the WBO 1976 led to a change in philosophy away from the assumption of an automatic acquisition of knowledge, skills and abilities through daily work practice towards an increased regulation with the specification of obligatory content and reference figures [5]. From 1968 onwards, structuring considerations were increasingly incorporated into the WBO. Various structuring concepts were introduced, renamed and in some cases discarded [1]. In 1992, the “subspecialties” were renamed “specialties” and so-called “specialist knowledge” (“Fachkunden”) and “optional specialty training” (“fakultative Weiterbildung”) were introduced in order to make it possible to teach the large areas in a manageable amount of time and to make special content optional [5]. Since 2003, in addition to further attempts at structuring, didacticization efforts have been evident for the first time in the WBO regulations, which have been further strengthened in the currently valid WBO 2018 [1]. The development of the WBO 2018 was already decided in 2010 by the mandate of the DÄT and confirmed two years later with the demand for a competence-based WBO [5], [6]. Subsequently, the “Committee for Postgraduate Education” (“Ständige Kommission Weiterbildung”, Stäko) developed the currently valid MWBO with representatives from the LÄK and the German Medical Association (“Bundesärztekammer”, BÄK), with the participation of the FG, professional associations, umbrella organizations and other medical organizations. The WBO 2018 has only been gradually implemented in hospitals since 2020, with some LÄKs not putting the new regulations into effect until 2022 [1]. The process of developing the new WBO has therefore taken over 10 years, which highlights the length and complexity of this process of change. The reluctance of the federal states to exert political influence on the WBO is sometimes critically assessed as* “quasi-complete state abstinence in matters of medical service planning and quality assurance”* and the resulting *“dysfunctional separation”* between the educational phases of undergraduate and postgraduate medical education is criticized [7].

With reference to the development of the WBO described above, this study uses the analysis of the adaptation of the WBO between 1992 and 2018 to examine to which extend the aspired changes can contribute to changing and didactically optimizing postgraduate education in hospitals.

## 2. Methods

The qualitative study described here is part of a more comprehensive study on postgraduate medical education using the example of surgery, which was conducted in 2019 [1]. An analysis of the formally defined structure for specialty training in the form of the MWBO was carried out. This was done both generally for specialty training and for specific aspects of surgery. A research design with a method triangulation [8] consisting of a non-reactive procedure (document analysis of the MWBO 1992, 2003 and 2018) and a supplementary reactive procedure (qualitative, guided expert interviews) was chosen in order to overcome the respective methodological limitations. The document analysis as the main method was carried out according to Mayring [9]. An analysis for differences was conducted, comparing the texts and content as well as analyzing word frequencies. This was done with the software MAXQDA 2020 (version 20.4.1). In addition, the development of the so-called “surgical catalogs” was examined, in which the reference figures for surgical interventions according to WB-RiLi were compared. The selection of the three MWBOs resulted from a consideration of the overall historical development, as these three regulations were subject to greater adjustments and, as indicated above, for the first time more significant structuring and didacticization approaches were incorporated into the MWBOs. In addition to the document analysis, expert interviews were conducted with stakeholders involved in the process to gain a deeper understanding of the changes made and the inherent system logic. This was not a case-by-case analysis, but rather a focus on expert status. One expert from the German Medical Association (BÄK) (103 min), one from State Chamber of Physicians (LÄK) (68 min) and one from the German Society for Surgery (FG) (59 min), who were actively involved in the process, were selected to cover the three relevant perspectives. Due to the selection of well-informed experts, the number of one person per perspective was considered sufficient. The interviews were audio-documented, transcribed with f4transcript (version 7.0.6) and analyzed with MAXQDA 2020 according to Kuckartz [10].

## 3. Results

In the word frequency analysis examining the terms used for the specialty training regulations, the use of the same eight terms in all three WBOs within the respective list of the 25 most frequent is revealed:

1. postgraduate education,
2. area,
3. therapeutic methods,
4. illness/diseases,
5. knowledge,
6. experience,
7. skills and
8. basics.

These terms thus appear to define the fundamental and temporally consistent character of specialty training, which the WBO is intended to reflect. The WBO is therefore about differentiation according to (medical) “areas”. It is also about learning “therapeutic methods” for various “illnesses/diseases” and thereby acquiring “knowledge”, “skills” and “experience”. This provides the “basics” for working as a specialist in the respective areas.

**Figure 1:**
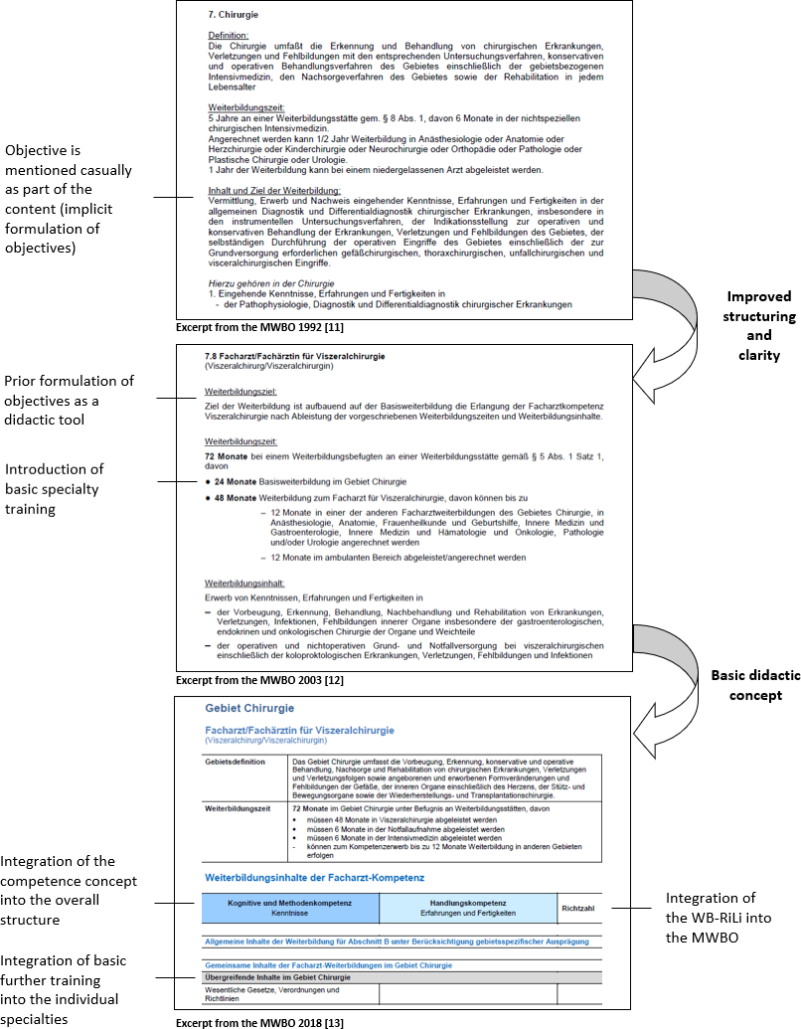
Visual comparison of the structure of the three MWBOs (1992, 2003 and 2018) and presentation of the adaptation

The document comparison with regard to structuring and didacticization revealed two major changes (see figure 1 (Fig. 1)). In the first step (from 1992 to 2003), the change can be classified as an improved textual clarity and more transparency as well as fundamental structural adjustments. An overview of these adjustments can be found in table 1 (Tab. 1). From 2003, the structural adjustment initiated the earliest possible specialization within the first specialty training. Since then, instead of first completing specialty training to become a surgeon and then acquiring a subspecialty, for example visceral surgery, specialty training in visceral surgery is completed immediately after licensing. Here, the number of years and requirements has been significantly reduced in the WBO. This also becomes apparent when comparing the mandatory reference figures between 1992 in the subspecialty “visceral surgery” with the figures that were stipulated from 2003 for specialty training in “visceral surgery” (see table 2 (Tab. 2)). The compulsory reference figures indicate a stronger structuring and regulation of the area of examination and surgical treatment methods that was already being applied in 1992. While no such specifications were made in 1987, these were explicitly defined with quantitative specifications from 1992 onwards. Even though these reference figures were greatly reduced again from 1992 to 2003, the structuring of this area remains in the figures. The reference figures for surgical interventions decreased from 1987 to 1992 and then only increased again in the area of general surgery from 2018. The comparability of the figures between 1992 and 2003 is somewhat limited overall, as the concept of basic specialty training was introduced, which was intended to cover the first two years of specialty training. This change must therefore be considered when interpreting the changed figures. In table 2 (Tab. 2), the reference figures for basic specialty training are inserted in an additional column (“basic”). These must be added to the figures for general surgery (in the table: “general”) or to the column for visceral surgery to enable comparability.

In retrospect, the BÄK puts the conceptual introduction of basic specialty training into perspective:

*“You know, the word basic specialty training is misleading. It was not BASIC specialty training. It was ALWAYS and that's how we defined it, but never put it in writing, it was the intersections between the individual specializations.”* (BÄK: 24)

In addition to the structural adjustment, the first didactic considerations in the transition from MWBO 1992 to MWBO 2003, such as the newly prescribed documentation obligation in so-called logbooks and annual specialty training dialogues, are also evident.

In the second step (from 2003 to 2018), the aim of the amendment, namely a fundamental reorganization with a focus on content and skills instead of time requirements with *“didactically adequate and appropriate* [reference figures]” [11] was implemented. In addition, the WBO 2018 was intended to further improve quality and transparency (e.g. with the help of the electronic logbook and specialty training plans). With the introduction of the concept of competence in the MWBO 2003, linguistic changes were made in this regard. Competence is defined for the first time under “definitions” (§2a) and then increasingly used (196 mentions, 12 of which are in the paragraph section, compared to the single mention in the MWBO 1992). This already shows the initial integration of the competence concept.

In the MWBO 2018, the use of competence terms expands significantly. In the newly introduced tabular presentation of specialist medical content (in section B), the various levels of competence (cognitive and methodological competence or operational competence) are presented as table columns (see figure 1 (Fig. 1)). This leads to a correspondingly more frequent use of the terms (competence: 70 mentions; operational competence, cognitive and methodological competence: 418 mentions each). The significant increase in the number of times these terms are used indicates the comprehensive integration of the competence concept. The competence-based approach should also be reflected in the use of reference figures:

*“(...) regardless of the reference figures, even if we have reference figures, they are not decisive. What is decisive is if the authorized person says he can do it now and if there are only 30, he can't do it after 90 times, then he normally doesn't get the checked mark.”* (BÄK: 44)

In the course of the amendment, consideration was given to abolishing the reference figures altogether. However, this was rejected by the physicians in specialty training: 

*“[There should be] no reference figures at all [anymore], because (...) competence cannot be tied to figures. But (...) the young residents were immediately beating [us] about the ears left and right, saying that we need the reference figures as a bargaining chip so that we can get certain performances there at all.”* (LÄK: 49)

The concept of basic specialty training was dropped again in the MWBO 2018. Instead, however, the overarching important specialty training content was added to the specialty training courses within the areas, so that the basic training should be integrated into every specialty training. This implementation of the conceptual consideration was achieved 

*“MUCH better in the CURRENT description of content”, which leads the BÄK to assume that this innovation “will be practiced more”* (BÄK: 24). 

On the other hand, when taking a fundamental look at the amendment in the expert interview, attention is drawn to the problems in the interaction between the requirements of the WBO and between the expectations of all those involved and the implementation in hospitals:

*“Well, we can specify what we want, but it has to be feasible in practice. (...) Whether it really happens in practice, we can only say when the first residencies are actually in training.”* (BÄK: 43)

The statements thus reveal a difference between the formal written rules of the WBO (completed) and the “lived” practice within the hospitals (not yet implemented). The LÄK mentions implementation problems in the form of “resistance[s]”. 

*“The resistance by persistence (...) [is] nowhere as great as in the medical profession.” *(LÄK: 93). 

In addition, the transfer of the MWBO into state law and the possible influence of the LÄK are viewed critically in some cases, which is made clear by the title “princes”, who would thus have power over the WBO:

*“And the German Medical Association then passes it on to the 17 State Chambers of Physicians and the princes can decide what they do. (...) They have completely different attitudes. And that is our shortcoming. And we must finally change that.”* (FG: 1)

The statements point to several obstacles in the communication between the stakeholders in medical self-administration and clinical practice, which limit opportunities for change and lead to potential conflict.

## 4. Discussion

The analysis of the WBO and the implemented changes revealed many attempts by the medical self-administration to design specialty training in hospitals closer to reality and didactically optimized. In the process, concepts occurred that were newly introduced and then removed again in the following WBO (specialist knowledge “Fachkunden”, optional specialty training, basic specialty training). Others were further developed, such as the introduction of the competence concept in the sense of competence-based postgraduate medical education [12]. The didactic instrument of the logbook for documenting training content was also improved. This was due to the presumed, often still inadequate use in hospital. The new electronic version is strongly linked to the operation and procedure code system (OPS system). The electronic logbook thus encourages continuous use rather than manual completion commonly conducted at the end of specialty training [1], which could have been a desired goal for better use in hospital. Finding a structure that makes sense in practice has been an implicit goal for decades (starting with the WBO 1968) [1], which can be seen to have been intensified once again in the three WBOs examined. 

The extent to which the structural adjustments and didactic design approaches of the WBO can influence specialty training practice in the hospitals depends on how the clinicians perceive the WBO and whether they accept it and thus make it “alive” or only tolerate it in some aspects. As has been shown in this study, eight terms are used consistently in the three WBOs examined. These terms thus reflect the fundamental, unchanged and recognized character of postgraduate education. Postgraduate education, defined as medical professional law that regulates the practice of the profession [13], is in turn a right that has been transferred by the state governments to the medical self-administration (represented by the LÄK). Accordingly, it can be assumed that a basic formal regulation of postgraduate education is also regarded as legitimate by clinicians. However, anything that goes beyond this basic regulation and attempts to actually implement an educational regulation with a didactic concept (an openly stated goal on the part of external training actors) risks losing its established, naturally given legitimacy and possibly being perceived as externally imposed. Innovations in the WBO have always been discussed controversially. All fundamental changes, such as the introduction of “optional specialty training” (“fakultative Weiterbildung”) for the WBO 1992 [14], the introduction of early specialization for the MWBO 2003 with basic specialty training or the competence concept of the WBO 2018, seem to be viewed critically by clinicians in the first place [1]. For the WBO 2018, which is only gradually being implemented in hospitals, an initial study shows that surgical trainers and trainees assume that the changes in the reference figures – more specification compared to the WBO 2003 as to what exactly can be accredited – mean that the specialist titles can no longer be achieved within the “previously usual time” and without overtime [15], which reflects a critical attitude on the part of the clinicians. This is particularly surprising because the definition of reference figures should actually be based on the proposals of the specialist societies and the declared aim of the new WBO was to focus more on the acquisition of competence than on fixed reference figures. Here, at least in the perception of clinicians, the goal may possibly not be achieved. In principle, the question must also be raised again here as to what extent the competence concept harmonizes at all with a continued mandatory specification of (minimum) reference figures. The current version of the WBO appears to be a compromise solution between a consistent implementation of the competency concept, in which a fixed number of procedures should no longer be considered equivalent to the acquisition of competencies, and the demand of many physicians in specialty training who had argued against the elimination of reference figures.

Overall, clinicians seem to have an ambivalent attitude towards the WBO, as the more comprehensive study on specialty training culture showed [1]. On the one hand, they express a desire for more structure, while on the other hand there is an increasing fear of external control. In principle, the path of medical self-administration with the WBO therefore appears to be the right one, even if steps have been taken forwards, sideways and back again through the introduction, adaptation and, in some cases, elimination of structural components. It remains important to compare theoretical concepts with the possibilities of implementation in practice, also with regard to the situation in hospitals with staff and time shortages.

## 5. Conclusion

This analysis has shown how the WBO has become more structured and didactically designed over the years. For the implementation of the competence-based WBO 2018 and future WBOs in hospitals, clinicians, who are the essential implementers of the WBO in hospitals, need to be included even more intensively. This should not only take place during the development of the WBO, but especially during its implementation in the hospitals themselves. In particular, trainers should be appropriately prepared for the implementation of didactic concepts such as competence-based training as an inherent part of daily training practice.

## Note

This article is essentially based on “Habitualisierung im ärztlichen Feld: Die fachärztliche Weiterbildung in Struktur und kultureller Praxis am Beispiel der Chirurgie” [Habitualization in the medical field. Postgraduate education in structure and cultural practice] [1]]. Permission by Springer Fachmedien Wiesbaden was granted for the republication of the content.

## Ethics

This project was conducted in accordance with the Declaration of Helsinki. Participation in the interview study was voluntary and the data was analyzed pseudonymously and anonymized for publication. Written declarations of consent were provided.

## Authors’ ORCIDs

- Sarah Prediger: [0000-0001-5483-1983]
- Daniela Rastetter: [0000-0002-5683-5459]
- Sigrid Harendza: [0000-0002-7920-8431]

## Acknowledgements

Special thanks to the interviewees, without whose willingness to speak openly with SP and provide insights into their perceptions, this study would not have been possible. We would also like to thank the German Medical Association for kindly providing the documents of the older MWBOs.

## Competing interests

The authors declare that they have no competing interests. 

## Figures and Tables

**Table 1:**
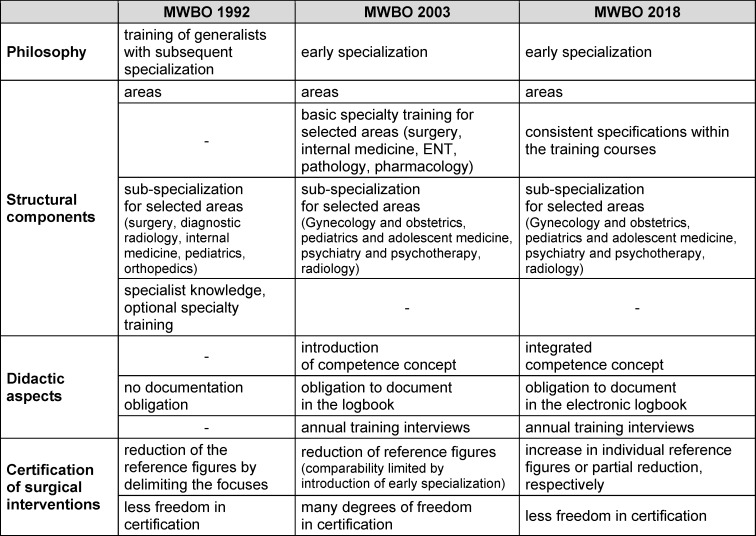
Summarized presentation of the most important changes between the MWBOs

**Table 2:**
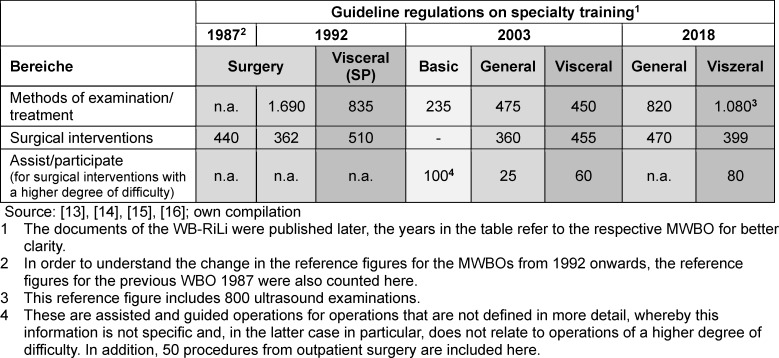
Comparison of the development of reference figures
